# Comparative Study on Growth and Metabolomic Profiles of Six Lactobacilli Strains by Sodium Selenite

**DOI:** 10.3390/microorganisms12101937

**Published:** 2024-09-24

**Authors:** Longrui Wang, Jiasheng Ju, Huichun Xie, Feng Qiao, Qiaoyu Luo, Lianyu Zhou

**Affiliations:** 1 Key Laboratory of Medicinal Plant and Animal Resources of the Qinghai–Tibetan Plateau in Qinghai Province, Xining 810008, China; 202232331032@stu.qhnu.edu.cn (L.W.); 2025090@qhnu.edu.cn (H.X.); 2025089@qhnu.edu.cn (F.Q.); 2014114@qhnu.edu.cn (Q.L.); 2 Academy of Plateau Science and Sustainability, Xining 810008, China; 3 School of Life Science, Qinghai Normal University, Xining 810008, China; 202032331031@stu.qhnu.edu.cn; 4 Food and Drug Testing Center, Xianyang 712000, China

**Keywords:** lactobacilli strains, selenium, metabolomic, metabolite analysis

## Abstract

Selenium (Se) has garnered increasing attention in the field of nutrition, as it is essential for both humans and animals. Certain microorganisms can enrich inorganic selenium and convert it into organic selenium. The growth and metabolomic profiles of six lactobacilli strains exposed to 50 μg/mL of sodium selenite were performed using gas chromatography tandem time-off light mass spectrometry (GC-TOF-MS) analysis. The addition of selenium significantly increased both the population and weight of the *Lacticaseibacillus rhamnosus* PS5, *Lbs. rhamnosus* RT-B, *Limosilactobacillus reuteri* 3630, and *Lmb. reuteri* 1663 strains, while those of the other two strains decreased. A total of 271 metabolites were determined, with their concentrations ranked from highest to lowest as follows: organic acids and derivatives, oxygen compounds, lipids and lipid-like molecules, and benzenoids. In certain groups, the concentrations of serine, aspartic acid, trehalose, palmitic acid, methylthreonine, and melibiose increased significantly, whereas glucuronic acid, ribose, ornithine, and methionine were downregulated. The metabolic pathways were significantly associated with ABC transporters, glycine, serine, threonine metabolism, and aminobenzoate degradation and other pathways. Based on these findings, we concluded that the transport, absorption, assimilation, and stress response to selenium by lactobacilli in metabolomic changed. Furthermore, the metabolomic alterations among different types of lactobacilli varied primarily due to their distinct properties.

## 1. Introduction

Selenium, an essential trace element, plays a crucial role in reproduction, immune function, growth regulation, development, and differentiation, as well as body metabolism. It is of utmost importance for human and animal health. Moreover, selenium possesses antioxidant and anti-cancer properties [1,2,3,4]. A deficiency in selenium can lead to over 40 diseases, including Keshan disease, Kaschh–Beck, cancer, cardiovascular disease, diabetes, and compromised immunity [5,6]. The World Health Organization recommends a dietary allowance of 30 to 40 µg/day of selenium [7]. Consequently, selenium supplements are becoming increasingly popular for meeting nutritional needs and chemoprevention. Inorganic selenium is highly toxic and has a low absorption rate. Some researchers have found that microorganisms such as *Saccharomyces*, *Bacillus*, *Chlorella*, and *Bifidobacterium* can enrich inorganic selenium and convert it into organic selenium [8,9,10,11,12]. Selenium replaces sulfur in cysteine to form selenocysteine (SeCys), which is incorporated into selenoproteins, allowing for absorption and utilization by animals [13]. Certain lactobacilli are regarded as a preferable option among microbial producers of selenium nanoparticles, having received the Qualified Presumption of Safety (QPS) status from the European Food Safety Authority [14]. These microorganisms have been used for a considerable time in the production of various foods and beverages, including yogurt, cheese, pickles, and fermented meats [15,16]. The metabolites produced by lactobacilli during fermentation, such as organic acids, amino acids, exopolysaccharides, and bacteriocins, exhibit a range of beneficial effects, including antioxidant activity, tumor inhibition, immune enhancement, bacteriostatic properties, antiviral activity, maintenance of intestinal flora balance, antidiarrheal effects, and antiallergenic properties [17,18,19,20].

Indeed, factors such as the initial concentration of selenium, the pH value of the growing environment, and the source of selenium significantly influence the enrichment ability of lactobacilli [14,21]. However, the classification of lactobacilli is a crucial factor affecting their capacity to enrich selenium [22], and different strains exhibit varying tolerances and accumulation capacities for selenium when cultivated in a selenium-rich environment. In a study conducted by Lamberti et al., it was revealed that the number of colonies decreased as the selenium concentration rose when *Lmb. reuteri* was cultivated in Na_2_SeO_3_ at concentrations ranging from 2.19 to 87.6 mg/L for 8 h [23]. Other studies evaluated the growth of *Lacticaseibacillus paracasei* subsp. *paracasei* CCDM 213 and *Bifidobacterium longum* subsp. *longum* CCDM 219 in the presence of 0–100 mg/L Na_2_SeO_3_ and found that both of the strains decreased with increasing concentrations of sodium selenite [24], yet the distinction lies in that they showed different downward trends over time. Recent investigations revealed that the microbial growth of *Lactiplantibacillus paraplantarum* and *Fructobacillus tropaeoli* was nearly the same when they were grown in the MRS-fructose with 5 mg/L of Se, but the selenium-enriching capacity of the latter was about 48.9% higher than that of the former. This is evidently attributed to the difference in the characteristics of the two strains as well [25].

Selenocysteine incorporation can effectively improve the enzymatic properties of lactobacilli through a genomic approach [26]. Wang et al. [27] elucidated the regulatory mechanisms involved in selenium enrichment in *Arthrospira platensis* by comparing the proteomic profiles of this strain before and after selenium enrichment. Other researchers analyzed the pathways of SeCys degradation via transcriptome analyses, providing insights into Se metabolism [28]. Furthermore, metabolomics methods can be employed to investigate the characteristics of selenium enrichment in lactobacilli, thereby enhancing our understanding of the biological effects and significance of Se-enriched *Lactobacilli bulgaricus* [15].

Over 50% of the organic selenium reduced by lactobacilli was found in the protein fraction, with 9.62–18.70% in the polysaccharide fraction and less than 0.75% in the nucleic acid fraction. Additionally, 20–30% of organic selenium was presented in other components, which possibly included lipids and various low molecular weight selenocompounds [12]. Selenium is primarily utilized by lactobacilli to produce selenocysteine, SeMet, and methyl selenocysteine, with selenocysteine being the predominant selenoprotein in selenium-rich yogurt [29,30]. Research conducted to elucidate the metabolic mechanism of Se-enriched lactic acid bacteria is scarce. Initially, Se oxyanions are transported into cells by non-specific transporter proteins (such as ABC transporter and sulphate permease). They are then gradually reduced to multiple intermediates (GS-Se-SG, GS-Se-) by substances such as GSH, nitrate reductase, and nitrite reductase. Ultimately, these intermediates are converted to Se^0^, which is subsequently assembled into SeNPs that are expelled from the cell [23].

Nuclear magnetic resonance, gas chromatography, and liquid chromatography (GC and LC) coupled with mass spectrometry (MS) are commonly employed to evaluate the metabolic profiles and their variations in lactobacilli strains, including amino acids, saccharides, lipids, and more, as well as the associated metabolic pathways. A recent study demonstrated that concentrations exceeding 6 mg/L of copper ions (II) significantly impact the metabolism of amino acids, lipids, the pentose phosphate pathway, and pyrimidine metabolism in *Lpb. plantarum* [31]. Other researchers investigated the metabolomic changes of *Lbs. casei* fermented in pineapple juice using NMR—based metabolomics and found a significant reduction in carbohydrate content, while the levels of lactic acid and γ-aminobutyric acid (GABA) increased [16]. Hu et al. [32] used the exopolysaccharides produced by *Pediococcus acidilactici* for yogurt fermentation; untargeted metabolomics analysis via UPLC-QTOF MS revealed 72 differential metabolites, including amino acids, nucleotides, and organic acids.

However, there is still a deficiency of information regarding the metabolic changes that occur in *Lbs. rhamnosus*, *Lactobacillus* sp., and *Lmb. Reuteri.* Currently, few methods have been used to study the mechanisms underlying the microbial metabolism of selenium. Gómez-Gómez et al. [33] gained valuable insights into the mechanism by which *Lmb. reuteri* CRL 1101 resists and biotransforms selenite into seleno amino acids using a quantitative proteomics approach. Other researchers have used comparative genomics to generate a detailed global species map of selenium utilization in bacteria by analyzing a large number of sequenced organisms.

The current study aims to deduce the metabolic changes that occurred in six different lactobacilli strains when exposed to 50 μg/mL sodium selenite at 37 °C for 36 h using a metabolomics approach. Investigating the metabolic mechanisms of selenium through the enrichment of selenium by various lactobacillus strains is essential.

## 2. Materials and Methods

### 2.1. Lactobacilli Strains

*Lbs. rhamnosus* LLR (St), *Lactobacillus* sp. JCM 7736 (LB), *Lbs. rhamnosus* PS5 (WX), *Lbs. rhamnosus* RT-B (RS), *Lmb. reuteri* 1663 (SS), and *Lmb. reuteri* 3630 (FG) were previously isolated from four types of commercially available yogurt by our team. These strains have been identified using 16S rDNA sequence analysis.

The strains were cultured on MRS media (pH 6.2–6.6) consisting of 1.0 g/L beef extract (BR, Shanghai Bowei Biotechnology Co., Ltd., Shanghai, China), 0.5 g/L yeast powder, 0.1 g/L peptone (BR, Beijing Aobox Biotechnology Co., Ltd., Beijing, China), 2.0 g/L dextrose (AR, Hongyan Chemical Reagent Factory, Tianjin, China), 0.005 g/L K_2_HPO_4_, 0.02 g/L MgSO_4_·7H_2_O, 0.005 g/L MnSO_4_·4H_2_O (AR, Shanghai Guangnuo Chemical Technology Co., Ltd., Shanghai, China), 0.1 g/L tween 80 (AR, Tianjin Kemiou Chemical Reagent Co., Ltd., Tianjin, China), 0.2 g/L ammonium citrate, and 0.5 g/L sodium acetate (AR, Tianjin DingShengXin Chemical industry Co., Ltd., Tianjin, China) and incubated at 37 °C for 24–36 h until the counts of lactobacilli strains approximately reached 10^8^ cfu/mL as the primary culture. These values were determined using microscopic examination with a 16 × 25 hemocytometer.

### 2.2. Se Accumulation of Lactobacilli Strains

An amount of 200 μL of preculture was added to 50 mL tubes containing 30 mL of MRS liquid medium supplemented with 0 and 50 μg/mL sodium selenite, respectively. The cultivation was inoculated at a temperature of 37 °C for 36 h. All experiments were carried out in triplicate.

### 2.3. Determination of the Quantity and Weight of Lactobacilli Strains

After inoculation, the counts of strains from cultures were determined using a hemocytometer with a size of 16 × 25. The bacteria suspension was centrifuged (2000× *g*, 5 min) and washed three times with 30 mL of sterile water. Subsequently, the pellet was dried in an oven at 60 °C until it reached a constant weight and was then weighed on an electronic balance.

### 2.4. Metabolites Extraction

After nitrogen drying, the pellet sample was weighed using the differential method. A mixture of methanol and chloroform (3:1, *v*/*v*) was added, along with L-2 phenylalanine (0.3 mg/mL) in proportion to the weight of the sample. The entire sample was treated with a 40 Hz grinding machine for 4 min and then ultrasonicated three times in an ice water bath for 5 min each. Following centrifugation (12,000× *g*, 4 °C, 5 min), 400 μL of the supernatant was transferred to a fresh 1.5 mL tube. To prepare the quality control (QC) sample, 200 μL from each sample was collected and combined.

After evaporation in a vacuum concentrator, 30 μL of methoxyamination hydrochloride (20 mg/mL in pyridine) was added and incubated at 80 °C for 30 min. The samples were then derivatized with 40 μL of bistrifluoroacetamide regent (1% trimethylsilyl, *v*/*v*) at 70 °C for 1.5 h. After gradually cooling the samples to room temperature, 5 μL of fatty acid methyl esters (FAMEs) in chloroform were added to QC sample. Three independent replicates of each treatment were prepared.

### 2.5. GC-TOF-MS Metabolite Profiling of Extracts

The derivatized extracts were analyzed using the Agilent-7890 GC (Agilent Technologies Inc., Santa Clara, CA, USA) coupled with Pegasus HT TOF-MS (LECO, Laboratory Equipment Co., Ltd., San Jose, CA, USA) and DB-5MS capillary column (Agilent Technologies Inc., Santa Clara, CA, USA). 1 μL aliquot of the derivatized sample was injected in splitless mode. Helium served as the carrier gas, with a front inlet purge flow rate of 3 mL/min and a gas flow rate through the column of 1 mL/min. The initial temperature was maintained at 50 °C for 1 min, then raised to 310 °C at a rate of 10 °C/min, where it was held for an additional 8 min. The temperatures for the injection port, transfer line, and ion source were set at 280, 280, and 250 °C, respectively. The mass spectrometer operated in electron impact mode at 70-eV, with an acquisition mass range of *m*/*z* 50–500 at a rate of 12.5 spectra per second, following a solvent delay of 6.25 min.

### 2.6. Data Processing and Statistical Analysis

The GC-MS data were imported into Chroma TOF (version 4.3x, LECO) software for peak extraction, baseline adjustment, deconvolution, alignment, and integration. The LECO-Fiehn Rtx5 database was used to characterize the metabolites by matching the mass spectrum and retention index. After screening, all peak signal intensities in each sample were normalized according to the internal standards, with less than half of the QC samples or RSD > 30% in QC samples. R software (version 4.4.0) was used to construct a heatmap of hierarchical clustering analysis and Venn analysis. PCA analysis was performed to visualize the metabolic differences among experimental groups using SIMCA (version 16.0.2) software. Metabolites meeting the predefined criteria of VIP (Variable Importance in Projection) ≥1 and *p* < 0.05 (Student’s *t*-test) were designated as differentially accumulated metabolites (DAMs). The differentially accumulated metabolites were annotated to the KEGG database (https://www.kegg.jp/), and the enrichment results of the metabolic pathways were displayed using the R package ggplot2 (version 3.3.5).

Using SPSS 26 software, Duncan’s multiple range tests were applied to compare significant differences (*p* < 0.05) among different trains. The population and weight of the same strain between the control and Se-treated groups were performed using a student’s *t*-test with a confidence interval at *p* < 0.05 (marked with *) and *p* < 0.01 (marked with **).

## 3. Results

### 3.1. The Effect of Selenium on the Population of Six Lactobacilli Strains

The changes in the population of six lactobacilli strains at a selenium concentration of 50 µg/mL are shown in Table 1. Among the six lactobacilli strains, the number of St and LB strains increased by approximately 9.43% and 14.20%, respectively, compared with the control group. In contrast, the number of WX, RS, FG, and SS strains decreased by approximately 7.94–16.06%.

Under conditions without selenium supplementation, the population of the SS strain was 5.08–121.30% higher than that of the other strains (*p* < 0.05). At a selenium concentration of 50 µg/mL, the population of the St strain was 9–112% higher than that of the other strains, including LB, WX, RS, FG, and SS (*p* < 0.05).

### 3.2. The Effect of Selenium on the Weight of Six Lactobacilli Strains

Table 2 shows the changes in the weight of six lactobacilli strains at selenium concentrations of 0 and 50 µg/mL. Compared with the control group, the cell weight of St and LB strains in the presence of Se increased by 7.87% and 14.29%, respectively. In contrast, the cell dry weight in the WX, RS, FG, and SS strains decreased by 10.42–16.01%.

When exposed to conditions without selenium addition, the SS strain exhibited the highest cell weight, while the LB strain demonstrated the lowest. At a selenium concentration of 50 µg/mL, the cell weight of the St strain was significantly higher than that of strains LB, WX, RS, FG, and SS (*p* < 0.05), exceeding by 9.09–123.26%.

### 3.3. Classification and Proportion of Metabolites Found in the Lactobacilli Strains

**Figure 1:**
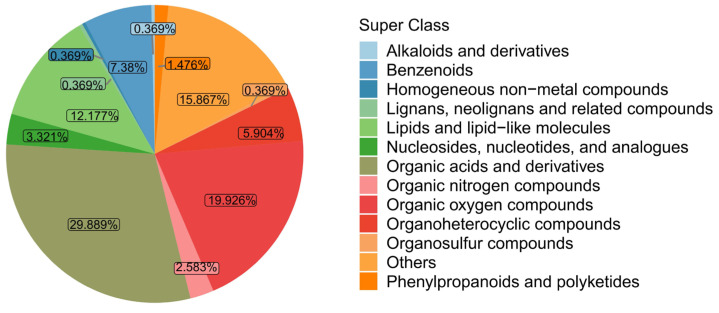
Pie plot of metabolite classification and proportion of metabolites found in the strain samples.

Qualitative and quantitative metabolomic analyses were performed on six strain samples. A total of 693 peaks were detected, and 642 were retained following a series of data management procedures. Subsequently, 271 metabolites were confirmed according to their chemical shifts and categorized into 12 distinct superclasses (Figure 1). These superclasses included 29.89% organic acids and derivatives; 19.93% organic oxygen compounds; 12.18% lipids and lipid-like molecules; 7.38% benzenoids; 5.90% organoheterocyclic compounds; 3.32% nucleosides, nucleotides, and analogues; 2.58% organic nitrogen compounds; 1.48% phenylpropanoids and polyketides; 0.37% alkaloids and derivatives; 0.37% homogeneous non-metal compounds; 0.37% lignans, neolignans, and related compounds; 0.37% organosulfur compounds; and 15.87% classified as others.

### 3.4. Principal Component Analysis of Lactobacilli Strains

PCA was conducted to visualize the overall changes in metabolites across all 12 samples. The first principal component accounted for 13.7% of the total variance, the second principal component explained 9.4%, and the third principal component contributed 8.7%. Together, these three components accounted for a total of 31.8% of the variance.

**Figure 2:**
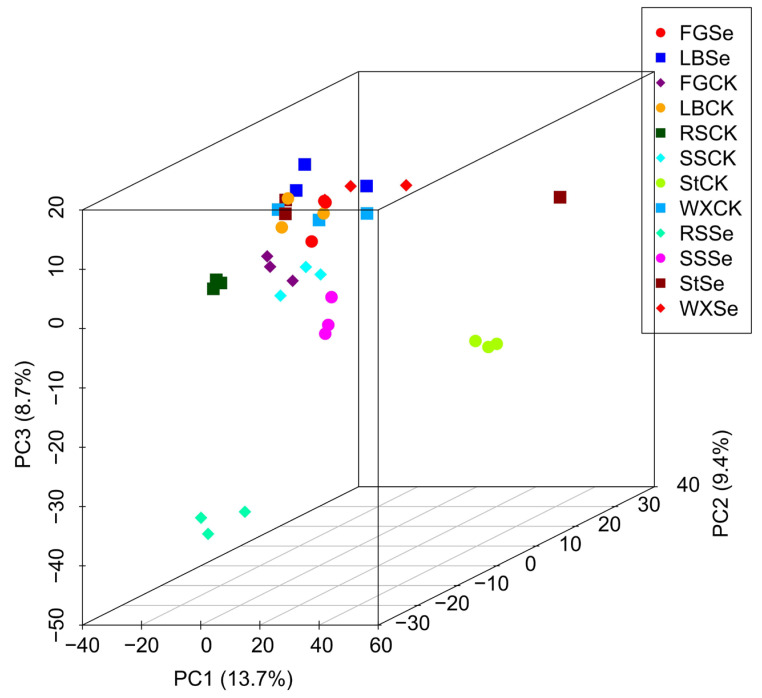
Principle component analysis (PCA) score plot of first, second, and third PCs from 12 lactobacilli strain samples. PC1: the first principal component; PC2: the second principal component; PC3: the third principal component. FGCK, LBCK, RSCK, SSCK, StCK, and WXCK represent strains grown without Se. FGSe, LBSe, RSSe, SSSe, StSe, and WXSe represent strains grown in the presence of selenium.

Figure 2 revealed distinct separations between the RSSe group and the RSCK group, the StSe group, and the StCK group, as well as a slight separation between the FGSe group and the FGCK group. In contrast, the LBSe group, SSSe group, and WXSe group were closely clustered with their respective control groups. In addition, the WXSe group, FGSe group, WXCK group, LBSe group, LBCK group, and StSe group were clustered together.

### 3.5. Heatmap and Hierarchical Cluster Analysis of Lactobacilli Strains

**Figure 3:**
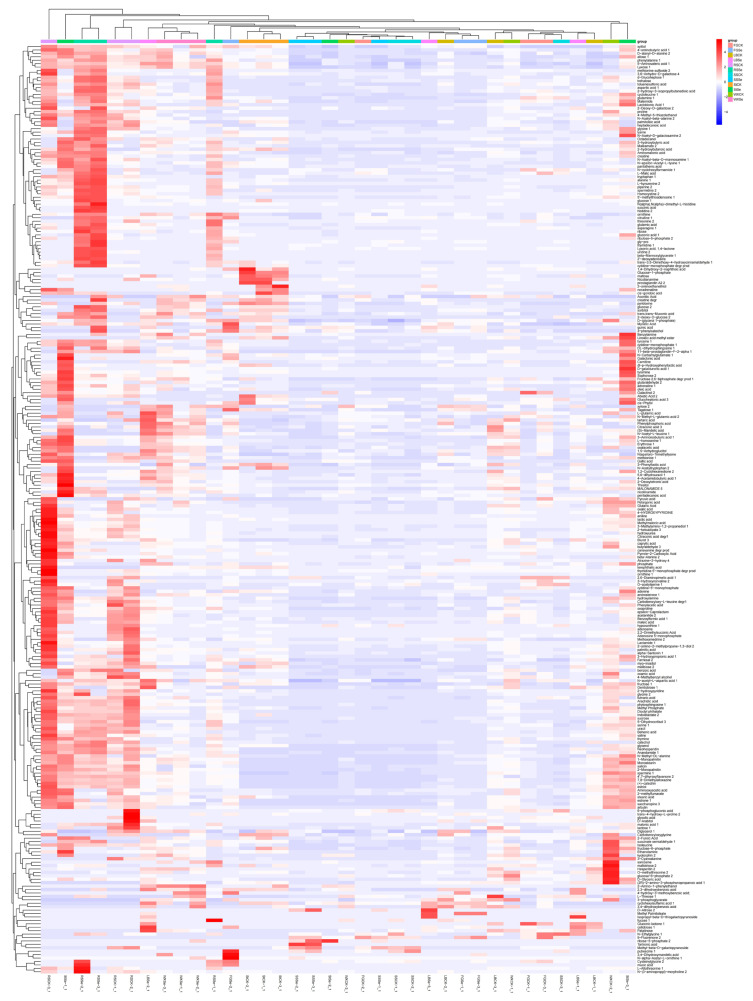
Heatmap and hierarchical cluster analysis for the 272 metabolites in the lactobacilli strain samples. The abscissa represents the different sample groups, the ordinate represents all metabolites, and the color blocks at different positions represent the correspondence and the relative expression of the metabolite at the location; red indicates high expression of the substance, and blue indicates low expression of the substance (see scale bar). RSCK-1_1, RSCK-2_1, RSCK-3_1, and RSSe-1_1, RSSe-2_1, RSSe-3_1 represent three parallel control groups without selenium addition and three 50 µg/mL selenium-added parallel control groups in the RS group, respectively, and so on.

The heatmap and hierarchical cluster analysis of all samples were used to identify the different metabolites between them (Figure 3). A total of 272 metabolites were identified, and the hierarchical clustering analysis indicated that there was good discrimination between samples. The samples were grouped into 12 categories (category 1–12). The 1st (RSCK-3), 2nd (StSe-1), 3rd (RSSe-2, RSSe-3), 4th (RSCK-1, RSCK-2), 5th (LBSe-2, WXSe-3, WXSe-1, WXSe-2), 6th (RSSe-1), 7th (FGSe-2), 8th (StCK-1, StCK-2, StCK-3), 11th (WXCK-2), and 12th (StSe-3) categories were clearly separated from the other categories. In contrast, the 9th category included 12 samples (SSSe-2, SSSe-3, StSe-2, WXCK-3, FGCK-2, SSSe-1, SSCK-1, SSCK-2, LBSe-3, LBCK-2, FGSe-1, FGSe-3), which did not exhibit significant separation among them. The 10th category included seven samples (LBCK-3, WXCK-1, FGCK-1, FGCK-3, SSCK-3, LBSe-1, LBCK-1), and these samples also could not be clearly separated from one another.

### 3.6. Marked Metabolites Obtained by Comparison before and after Selenium Addition

**Figure 4:**
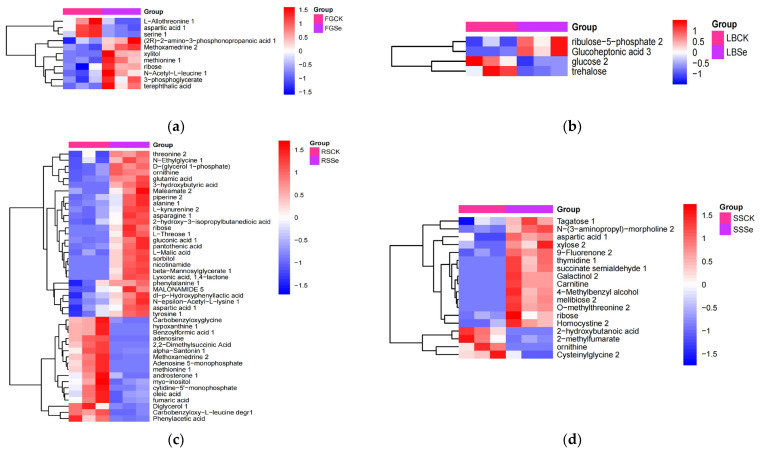

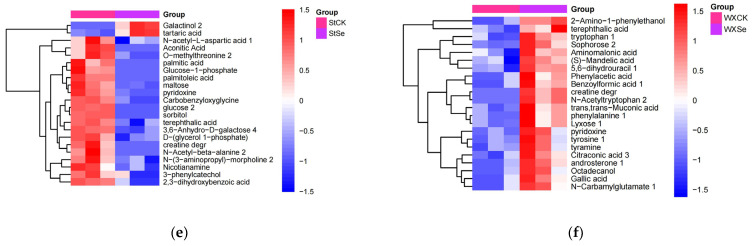
Marked metabolites in heatmap of hierarchical clustering analysis for groups without selenium (CK) vs. 50 µg/mL selenium-added groups (Se). Red and blue blocks indicate higher and lower metabolite levels, respectively (see scale bar). The subfigures (**a**–**f**) represent the results before and after the addition of selenium comparison of the strains of FG, LB, RS, SS, St, and WX, respectively.

**Figure 5:**
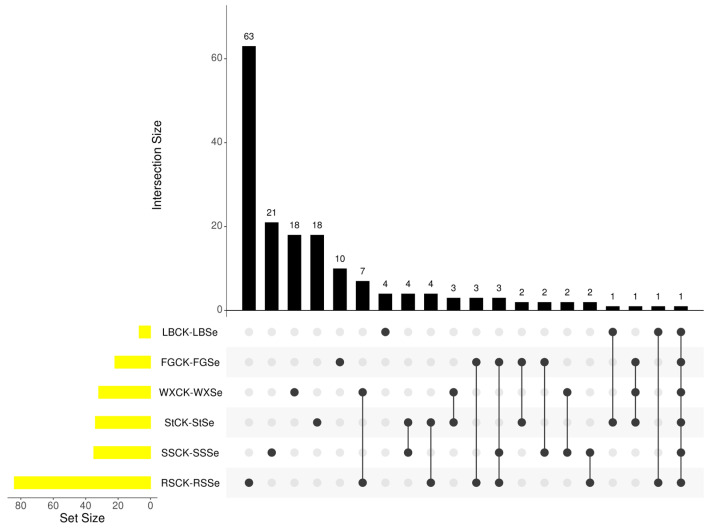
Venn analysis for six strains with and without Se. Each dot represents a group, and the Set Size corresponding to each dot represents the number of metabolites contained in the group. The dot connections corresponding to the abscissa of the column chart represent the comparisons of the groups, and the ordinate shows the number of differential metabolites shared by each group. LBCK-SSLB, FGCK-FGSe, WXCK-WXSe, StCK-StSe, SSCK-SSSe, and RSCK-RSSe represent the comparison of the results before and after the addition of selenium for the strains of LB, FG, WX, St, SS, and RS, respectively.

Differentially accumulated metabolites (DAMs) identified by comparison before and after selenium addition, which can also be considered as candidate biomarkers, were selected based on VIP > 1 and *p* < 0.05. Consequently, there were 11 DAMs in the FGCK-FGSe group when compared with the CK group, the content of six DAMs rose, while three DAMs reduced (Figure 4a). In the LBCK-LBSe group, we observed only four DAMs, the content of glucoheptonic acid increased by 39.65 times, ribulose-5-phosphate increased by 6.14 times, and glucose and trehalose reduced to 0.19 and 0.15 times, respectively (Figure 4b). In the RSCK-RSSe group, there were 44 DAMs, with 27 being upregulated and 17 downregulated. Notably, six DAMs increased by more than 8 × 10^3^ times (Figure 4c). In the SSCK-SSSe group, 18 DAMs increased to 1.61–23.19 times, and seven DAMs increased to 5 × 10^4^–3 × 10^5^ times, while four DAMs reduced to 0.17–0.35 times (Figure 4d). We also observed 22 DAMs in the StCK-StSe group, with only two DAMs increasing and 20 decreasing; it is worth noticing that the content of galactinol increased by 4.6 × 10^5^ times (Figure 4e). Moreover, in the WXCK-WXSe group, only two DAMs increased, while 20 DAMs reduced; specifically, the content of creatine increased by 1.2 × 10^4^ times, and N-acetyltryptophan increased by 1.4 × 10^4^ times (Figure 4f). The six strains shared one metabolite (Figure 5). In addition, the unidentified compounds among the DAMs were involved in the strains’ response to selenium. Overall, the types of DAMs and their magnitude of change varied across different groups.

### 3.7. Key Marked Metabolites Change

#### 3.7.1. Amino Acids and Derivatives

In the FGSe group, the concentrations of L-allothreonine, aspartic acid, and serine were lower than in the CK group, although (2R)-2-amino-3-phosphonopropanoic acid, methionine, and N-acetyl-L-leucine increased. In the RSSe group, the concentrations of 10 DAMs including threonine, ornithine, glutamic acid, alanine, kynurenine, asparagine, phenylalanine, N-epsilon-acetyl-L-lysine, aspartic acid, and tyrosine were upregulated, whereas carbobenzyloxyglycine, methionine, and carbobenzyloxy-L-leucine were downregulated, with methionine’s concentration notably decreasing to 5.44 × 10^−4^ times. The SSSe group showed increased concentrations of aspartic acid, O-methylthreonine, and homocystine, with O-methylthreonine reaching over 5 × 10^5^ times. However, ornithine and cysteinyl glycine were downregulated to 4.27 × 10^−7^ and 0.17 times, respectively. For the StSe group, the concentration of N-acetyl-L-aspartic acid, O-methylthreonine, and N-acetyl-beta-alanine were downregulated, with N-acetyl-beta-alanine decreasing to 6.16 × 10^−7^ times. In addition, in the WXSe group, the concentrations of tryptophan, phenylalanine, tyrosine, and N-carbamylglutamate were upregulated.

#### 3.7.2. Organic Acids and Derivatives

Compared to the control groups, the FGSe group showed an increase in the concentration of terephthalic acid, while the LBSe group demonstrated a remarkable upregulation of glucoheptonic acid (39.65 times). Several DAMs of the RSSe group were significantly upregulated, including 3-hydroxybutyric acid, 2-hydroxy-3-isopropylbutanedioic acid, gluconic acid, pantothenic acid, L-malic acid, phenylalanine, and dl-p-hydroxyphenyllactic acid. The 3-hydroxybutyric acid showed a considerable rise, exceeding over 5 × 10^5^ times. Conversely, benzoylformic acid, 2,2-dimethylsuccinic acid, fumaric acid, and phenylacetic acid were downregulated, with the first two decreasing to 1.72 × 10^−6^ and 1.29 × 10^−8^ times, respectively. In the SSSe group, the concentration of 2-hydroxybutanoic acid was downregulated to 3.31 × 10^−6^ times. Concerning the StSe group, the concentration of tartaric acid was upregulated, while aconitic acid, terephthalic acid, and 2,3-dihydroxybenzoic acid were downregulated, with the former decreasing to 9.36 × 10^−5^ times. Lastly, in the WXSe group, increases in the concentration of terephthalic acid, aminomalonic acid, mandelic acid, phenylacetic acid, benzoylformic acid, acetyltryptophan, trans-muconic acid, citraconic acid, and gallic acid were observed.

#### 3.7.3. Carbohydrates and Their Analogues

Compared to the control groups, the LBSe group exhibited an increase in the concentration of glucoheptonic acid, while the RSSe group showed an upregulation of gluconic acid. In the SSSe group, tagatose, xylose, and melibiose, were upregulated, with melibiose increasing to over 7 × 10^5^ times. In contrast, the StSe group experienced a down-regulation of glucose-1-phosphate, maltose, glucose, and 3,6-anhydro-D-galactose, with glucose-1-phosphate and glucose decreasing to 5.07 × 10^−5^ and 3.62 × 10^−7^ times, respectively. In addition, the WXSe group showed an upregulation of sophorose and lyxose.

#### 3.7.4. Lipids and Lipid-like Molecules

Compared to the control groups, the concentration of 3-phosphoglycerate was up-regulated to 2.39 times in the FGSe group. In the RSSe group, D-(glycerol 1-phosphate), and beta-mannosylglycerate were upregulated, with the latter increasing to over 6 × 10^5^ times. Conversely, oleic acid and diglycerol were downregulated. The concentration of 2-methylfumarate was downregulated to 0.35 times in the SSSe group. Regarding the StSe group, the concentrations of palmitic acid, palmitoleic acid, and D-(glycerol 1-phosphate) were downregulated, with the former two reduced to 7.12 × 10^−8^ times and 8.13 × 10^−6^ times, respectively.

#### 3.7.5. Nucleosides, Nucleotides, Purines, Pyrimidines, Ribose, and Analogues

Compared to the control groups, the concentration of ribose increased in the FGSe, RSSe, and SSSe groups. In the RSSe group, the concentrations of cytidine-5′-monophosphate, adenosine, and hypoxanthine reduced to 0.06, 2.15 × 10^−5^, and 1.34 × 10^−5^ times, respectively. In the SSSe group, the concentration of thymidine was upregulated to over 7 × 10^5^ times, which is noteworthy. Additionally, in the WXSe group, the concentration of 5,6-dihydrouracil was also upregulated.

### 3.8. KEGG Enrichment Analysis of Metabolic Pathway Enrichment

**Figure 6:**
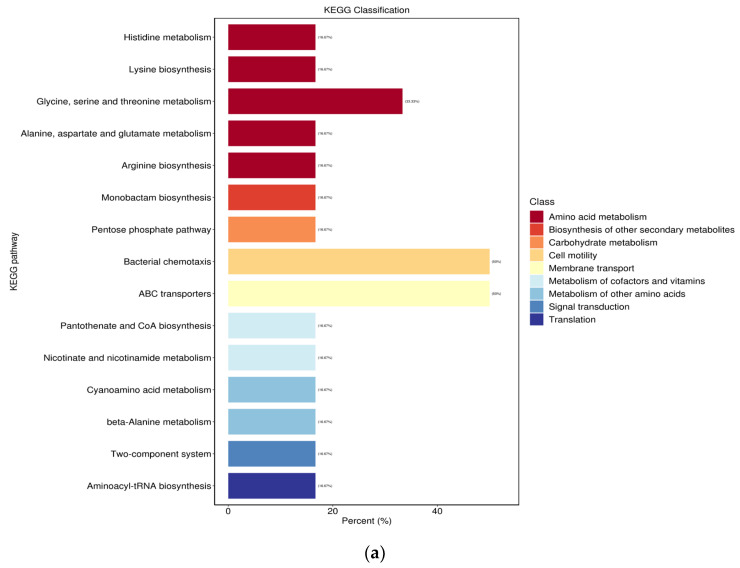

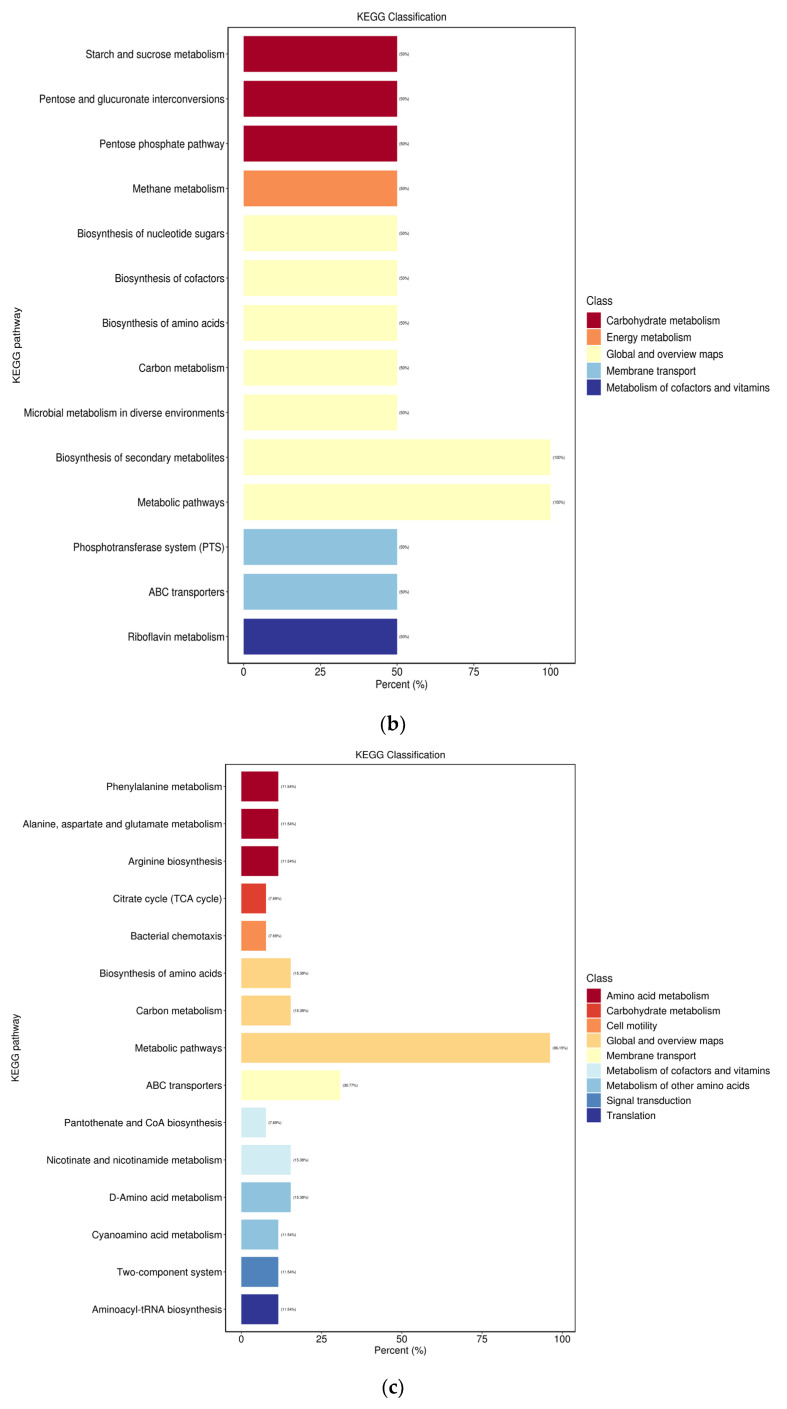

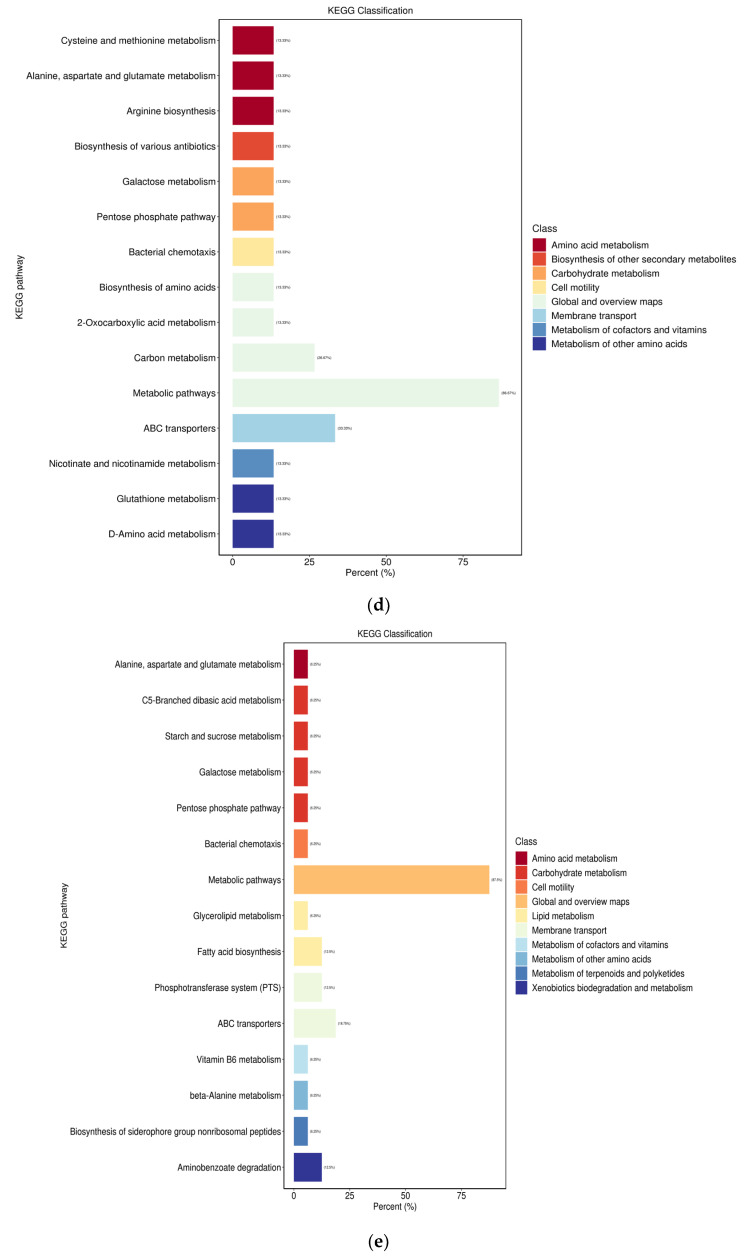

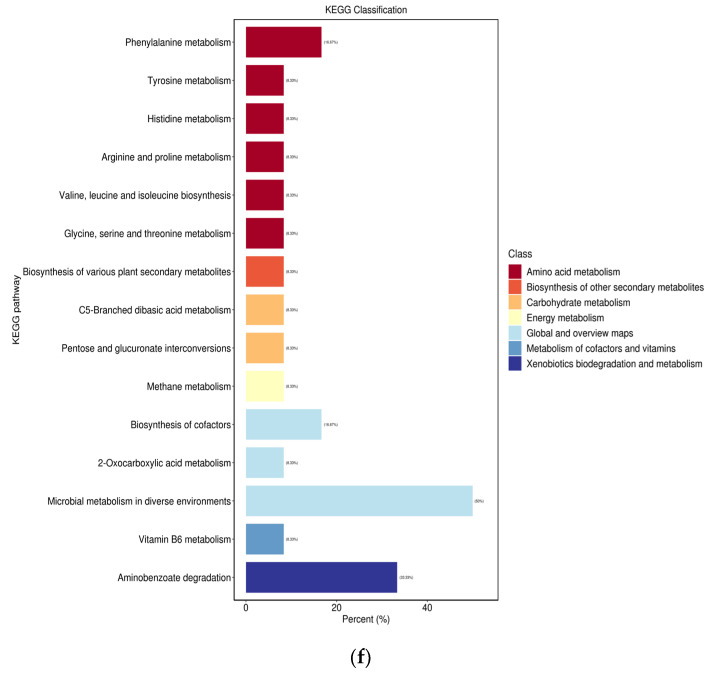
KEGG classification for group FGCK vs. FGSe (**a**), LBCK vs. LBSe (**b**), RSCK vs. RSSe (**c**), SSCK vs. SSSe (**d**), StCK vs. StSe (**e**), and WXCK vs. WXSe (**f**) in sequence. The abscissa represents the percentage of the number of annotated differential metabolites in a pathway compared to the number of all annotated differential metabolites, and the ordinate represents the name of the enriched KEGG metabolic pathway.

The Kyoto Encyclopedia of Genes and Genomes (KEGG) enrichment analysis of metabolic pathway enrichment (Figure 6) was conducted based on DAMs of the samples. Among the groups FGCK-FGSe, SSCK-SSSe, LBCK-LBSe, and RSCK-RSSe, the metabolic pathways were significantly associated with ABC transporters. Notably, only the FGCK-FGSe group demonstrated a strong relationship with glycine, serine, and threonine metabolism. Furthermore, only the WXCK-WXSe group exhibited a significant association with aminobenzoate degradation. Therefore, we conclude that the selenium treatment has a substantial impact on amino acid metabolism, ABC transporters, and aminobenzoate degradation.

#### 3.8.1. ABC Transporters

Based on the DAMs, aspartic acid and D-ribose appeared simultaneously in the ABC transporters pathway of the FGCK-FGSe, SSCK-SSSe, and RSCK-RSSe groups. The content of aspartic acid reduced to about 0.21 and 0.53 times in the SSCK-SSSe and RSCK-RSSe groups, but it increased by about 3.10 times in the FGCK-FGSe group. The content of D-ribose reduced to about 0.06–0.62 times across these three groups. L-ornithine appeared simultaneously in the ABC transporters of the SSCK-SSSe and RSCK-RSSe groups. It is worth noting that the content of L-ornithine increased to 2 × 10^6^ times in the SSCK-SSSe group but reduced to about 0.21 times in the RSCK-RSSe group. Additionally, melibiose, carnitine; alpha-trehalose; xylitol; myo-inositol, D-sorbitol, sn-glycerol 3-phosphate, L-threonine, and adenosine were exclusively found in the SSCK-SSSe, FGCK-FGSe, LBCK-LBSe, and RSCK-RSSe groups, respectively.

#### 3.8.2. Glycine, Serine, Threonine Metabolism and Other Pathways

Aspartic acid was also involved in glycine, serine, and threonine metabolism as DAM. Apart from this, the content of allothreonine increased by 3.42 times. Terephthalic acid, mandelic acid, gallic acid, and benzoylformic acid were involved in the aminobenzoate degradation pathway as DAMs. Their contents reduced by approximately 0.65, 0.48, 0.16, and 0.14 times, respectively.

## 4. Discussion

The fermentation of six different strains of lactobacilli in sodium selenite caused notable metabolomic changes. Various amino acids, organic acids, carbohydrates, and nucleotides, along with their related metabolic pathways, exhibited significant alterations, with each strain demonstrating unique changes.

Serine residues serve as the starting point for the selenium metabolism in bacteria [34]. They offer the carbon skeleton necessary for the synthesis of selenocysteine, making serine-phosphate-RNA a precursor for the formation of SeCys [35]. Our study found that the concentration of serine in the FGCK-FGSe group was much higher (by 7.0 times), which appears to be unique to this group. Serine enhanced the production of seleno-amino in *Streptococcus thermophilus*. Furthermore, during the biotransformation of inorganic selenium to organic selenium, the initial amino acid concentration increases alongside selenite [36]. To some extent, this conclusion, which aligns with our experimental results, may explain why the concentration of serine in FGCK-FGSe increased. The mechanism may establish the incorporation of selenium into proteins as a form of metabolized SeCyc by genetic coding [37]. In addition, DAMs were enriched in ABC transporters, suggesting that the addition of serine can promote the growth rate of *S. thermophilus* during the production of selenium-rich dairy products. Moreover, considering that SeCys-containing proteins were released into the environment by cells, the use of probiotic strains as nutraceuticals offered enhanced value [36,38]. Furthermore, the upregulation of serine concentration may indicate a stress response in lactobacilli strains exposed to selenium, and the overexpression of a serine protease could degrade misfolded proteins originating after selenite stress exposure in *Lactococcus lactis* [38].

Aspartic acid as a DAM and the pentose phosphate pathway occurred almost simultaneously in the SSSe-SSCK, FGSe-FGCK, and RSSe-RSCK groups. Aspartic acid is known to be a substrate of the gluconeogenesis pathway. Segura et al. [39] documented that aspartate was utilized by enterohemorrhagic *E. coli* as carbon and/or nitrogen sources. Notably, only the RSSe-RSCK and SSSe-SSCK groups consumed aspartic acid in our experiment, which may be related to the two strains’ varying environmental adaptation. Glutathione has been shown to play a crucial role in the detoxification of reactive metabolites. Glutathione is converted to its oxidized form (L-glutathione oxidized, GSSG) during this detoxification process. The concentration of GSSG is regulated by the effective action of the glucocorticoid receptor, which requires electrons primarily derived from NADPH, sourced mainly from the pentose phosphate pathway. In addition, it is a key source of electrons for many antioxidants [40]. Furthermore, the pentose phosphate pathway serves as a significant source of NADPH [41], suggesting its involvement in the detoxification of reactive metabolites in selenium-rich environments. This finding supports the potential for microorganisms to convert glutathione and utilize antioxidant enzymes, particularly in the context of industrial production of microorganisms as dietary supplements enriched with selenium.

The presence of selenium can lead to the excretion of polysaccharides while simultaneously forming a defense mechanism that enhances the ability of *Lmb. reuteri*, *L. acidophilus*, and *Lab. delbrueckii* subsp. *bulgaricus* strains to survive under stressful conditions [38,42]. Similarly, we found that the trehalose content in the LBSe-LBCK group increased by more than six times. When the bacteria are exposed to a harsh environment, trehalose in the cell will increase significantly, which is wrapped on the surface of the cell in the form of a protective film to protect the protein molecules from denaturation and inactivation, thus playing a protective role in the organism. A significant decrease in glucuronic acid content (reduced by 0.03 times) may also mean the formation of polysaccharides. In contrast, in the DAMs of the other groups, there was no notable change in the concentration of saccharide. In addition, our previous research indicated that the biomass and number of cells, as well as the levels of iron and potassium in the LBSe-LBCK group were markedly lower than those in the other groups when exposed to 50 μg/mL of sodium selenite. Furthermore, the phosphorus concentration in the cells of this group was elevated compared to that in the other groups, and DAMs exhibited significant enrichment in the phosphotransferase system (PTS). These findings indicated that the LBSe-LBCK group may have experienced the highest level of selenium stress. Exopolysaccharides produced by lactobacilli have prebiotic effects. They can modulate the human gut flora [43], so the stress state in the selenium environment and the production of different saccharides and polysaccharides may serve as criteria for probiotics.

In our experiments, we observed that a significant amount of palmitic acid was synthesized in the StSe-StCK group (over 1.4 × 10^7^ times). In contrast to the findings from the LBSe-LBCK group, the StSe-StCK group exhibited the highest values in terms of number, weight, iron content, selenium content, and selenium enrichment rate among all six strains. In *S. plymuthica*, the levels of C16:1 and C18:1 fatty acids in the cell membranes decreased, which reduced cell fluidity and increased hydrophobicity in response to selenium stress [29,44]. Zhai et al. [45] reported an upregulation of the acetyl-CoA carboxylase biotin carboxyl carrier protein subunit involved in the biosynthesis of fatty acids in the presence of selenium, leading to changes in the membrane of *Lpb. plantarum* when subjected to stress factors. Therefore, in the StSe-StCK group, *Lbs. rhamnosus* LLR-L2 likely altered the hydrophobicity of its cell membrane by increasing the content of palmitoleic acid, thereby coping with selenium stress. This adaptation reduces the strain’s stress and helps maintain its functionality as a probiotic.

In all six groups, ribose levels decreased significantly compared to the control group. It has been suggested that ribose can be directly consumed by the cell. Moreover, it was supported by studies demonstrating that the addition of ribose as an extra carbon source enhances the sporulation of *Bacillus subtilis* [46]. This discovery suggests that ribose may function as a viable carbon source. It was also observed that selenium nanoparticles were overexpressed as an enzyme when *Lmb. reuteri* was grown in the presence of selenium nanoparticles. This enzyme catalyzes the phosphorylation of ribose to D-ribose-5-phosphate, which is subsequently utilized for nucleotide synthesis in the pentose phosphate pathway [33]. In both the FGSe-FGCK and SSSe-SSCK groups, nucleic acids were consumed in small amounts. Simultaneously, DAMs were enriched in the pentose phosphate pathway, which aligns with the aforementioned results and suggests that the reduction of nucleic acids may also contribute to nucleotide synthesis.

Cell membranes are impermeable to selenium ions; therefore, cells must accumulate selenium through active transport. This capability is associated with the selenium storage capacity of lactobacilli in a selenium-rich environment, which influences the residual selenite levels during the fermentation process of selenium-enriched food products [47]. Five of the lactobacilli strains were linked to the ABC transporters. Fernando et al. [47] found an upregulation of amino acid and zinc ABC transporters in the presence of 5 ppm of Se, which appeared to be in agreement with our results. The DAMs were enriched in the ABC transport pathway as well as the pentose phosphate pathway and phosphotransferase system. Moreover, it has been claimed that arginine-tRNA and ABC transporters were up-regulated in the presence of selenite [33]. Consistent with this, the results we came up with suggested that Se-containing protein synthesis occurred in the presence of selenium during our experiment.

The phosphotransferase system (PTS) plays a vital role in the phosphorylation of various sugars and their derivatives, facilitating their transport into lactobacilli strains [41]. In *Fructilactobacillus tropaeoli*, the cellobiose-specific PTS IIC component, which is capable of converting sucrose into fructooligosaccharides, was repressed in the presence of Se [47]. This finding aligned with the results obtained in our experiment and might be linked to the stress induced by selenium or other metals. Bacteria also regulate their resistance and adaptation to environmental factors through the PTS. Therefore, it is possible that lactobacilli can modulate their tolerance to selenium-rich environments via this system. This capability is also related to the selenium storage capacity of lactobacilli and should be considered in the production of selenium-enriched foods.

## 5. Conclusions

Metabolic data were utilized to evaluate the differences before and after the addition of selenium of six different lactobacilli strains. Following the addition of selenium, the number and weight of four strains increased significantly, while the other two strains exhibited a decrease. The differential metabolites identified by comparing the conditions before and after selenium addition in *Lmb. reuteri* 3630 were primarily amino acids. In contrast, *Lactobacillus* sp. JCM 7736 predominantly showed changes in carbohydrates. For *Lbs. rhamnosus* RT-B, *Lbs. rhamnosus* PS5, and *Lbs. rhamnosus* LLR, the main metabolites were amino acids and organic acids, whereas *Lmb. reuteri* exhibited both amino acids and carbohydrates as the primary metabolites. Selenium had a pronounced effect on the biotransformation of organic selenium in *Lmb. reuteri* 3630. The addition of selenium influenced the gluconeogenesis pathway and detoxification processes in three strains: *Lmb. reuteri* 1663, *Lbs. rhamnosus* RT-B, and *Lactobacillus* sp. JCM 7736, the last strain might face the greatest selenium stress. To mitigate selenium stress, *Lbs. rhamnosus* LLR increased the content of palmitoleic acid in the cell membrane. Furthermore, the addition of selenium had a more significant impact on the ABC transporter in all five strains, with the exception of *Lbs. rhamnosus* PS5.

## Figures and Tables

**Table 1:** Effects of selenium concentrations on the population of six lactobacilli strains.

Concentration of Selenium (µg/mL)	The Population of Bacterial Cells × 10^8^ (cfu/mL)					
	St	LB	WX	RS	FG	SS
0	3.18 ± 0.06 ^C^	1.69 ± 0.02 ^F^	1.89 ± 0.03 ^E^	3.07 ± 0.05 ^D^	3.55 ± 0.03 ^B^	3.74 ± 0.03 ^A^
50	3.48 ± 0.06 ^A^ **	1.93 ± 0.01 ^E^ **	1.74 ± 0.02 ^F^ **	2.63 ± 0.02 ^D^ **	2.98 ± 0.04 ^C^ **	3.20 ± 0.03 ^B^ **

Note: Data represent means ± standard deviation (SD). *p* < 0.01 is marked with **. Uppercase letters on the same line indicate significant differences between different strains at the same concentration (*p* < 0.05). St, LB, WX, RS, FG, and SS respectively represent the growth conditions of the six strains mentioned above under different selenium concentrations: *Lbs. rhamnosus* LLR (St), *Lactobacillus* sp. JCM 7736 (LB), *Lbs. rhamnosus* PS5 (WX), *Lbs. rhamnosus* RT-B (RS), *Lmb. reuteri* 1663 (SS), and *Lmb. reuteri* 3630 (FG).

**Table 2:** Effects of selenium concentrations on the weight of six different lactobacilli strains.

Concentration of Selenium (µg/mL)	The Weight of Bacterial Cells (mg/mL)					
	St	LB	WX	RS	FG	SS
0	0.89 ± 0.02 ^C^	0.42 ± 0.01 ^F^	0.48 ± 0.01 ^E^	0.84 ± 0.00 ^D^	1.00 ± 0.01 ^B^	1.04 ± 0.01 ^A^
50	0.96 ± 0.02 ^A^ *	0.48 ± 0.01 ^E^ **	0.43 ± 0.00 ^F^ **	0.71 ± 0.01 ^D^ **	0.84 ± 0.02 ^C^ **	0.88 ± 0.01 ^B^ **

Note: Data represent means ± standard deviation (SD). *p* < 0.05 is marked with *, and *p* < 0.01 is marked with **. Uppercase letters on the same line indicate significant differences between different strains at the same concentration (*p* < 0.05). St, LB, WX, RS, FG, and SS respectively represent the growth conditions of the six strains mentioned above under different selenium concentrations.

## Data Availability

The data presented in this study are available within the article.
